# Multivariate space‐time modelling of multiple air pollutants and their health effects accounting for exposure uncertainty

**DOI:** 10.1002/sim.7570

**Published:** 2017-12-04

**Authors:** Guowen Huang, Duncan Lee, E. Marian Scott

**Affiliations:** ^1^ School of Mathematics and Statistics University of Glasgow Glasgow G12 8SQ UK

**Keywords:** air pollution and health, multiple pollutant fusion modelling, space‐time modelling, uncertainty propagation

## Abstract

The long‐term health effects of air pollution are often estimated using a spatio‐temporal ecological areal unit study, but this design leads to the following statistical challenges: (1) how to estimate spatially representative pollution concentrations for each areal unit; (2) how to allow for the uncertainty in these estimated concentrations when estimating their health effects; and (3) how to simultaneously estimate the joint effects of multiple correlated pollutants. This article proposes a novel 2‐stage Bayesian hierarchical model for addressing these 3 challenges, with inference based on Markov chain Monte Carlo simulation. The first stage is a multivariate spatio‐temporal fusion model for predicting areal level average concentrations of multiple pollutants from both monitored and modelled pollution data. The second stage is a spatio‐temporal model for estimating the health impact of multiple correlated pollutants simultaneously, which accounts for the uncertainty in the estimated pollution concentrations. The novel methodology is motivated by a new study of the impact of both particulate matter and nitrogen dioxide concentrations on respiratory hospital admissions in Scotland between 2007 and 2011, and the results suggest that both pollutants exhibit substantial and independent health effects.

## 1 INTRODUCTION

Air pollution continues to be a global public health problem, with a recent World Health Organisation report^1^ estimating that outdoor air pollution was responsible for the premature deaths of 3 million people under the age of 60 in 2012. In the United Kingdom, compared to the United Kingdom's single biggest killer, coronary heart disease, which kills nearly 23 000 people^2^ under the age of 75, an estimated 40 000 premature deaths are attributed to air pollution each year,^3^ making it one of the most substantial public health problems of our generation. Two types of adverse air pollution effects are typically estimated in the literature: short‐ and long‐term effects. Short‐term effects are effects observed immediately (within a day or so) after a high pollution episode and are estimated by regressing daily counts of disease cases against air pollution concentrations on the preceding few days using an ecological (at the population level) time series design. In contrast, the long‐term effects of pollution are effects resulting from prolonged exposure over months and years, and this type of effect is the focus of this study. Such long‐term effects can be estimated by cohort studies such as Laden et al,^4^ but they are expensive and time consuming to implement due to the long‐term follow up required for the cohort. Therefore, a small‐area spatio‐temporal ecological‐level study design can also be used, which uses routinely available data, and examples include Elliott et al,^5^ Lee et al,^6^ and Blangiardo et al.^7^

The disease data for this study design are counts of the total disease burden from the populations living in nonoverlapping areal units for consecutive time periods, and Poisson log‐linear models accounting for spatio‐temporal autocorrelation are typically used for the analysis. The pollution data available to characterise exposure comprise point‐level measurements from a network of monitors and output from atmospheric dispersion models, and both of these data types have been used to estimate pollution concentrations in existing epidemiological health studies. For example, point‐level monitored data are used by Elliott et al,^5^ while modelled concentrations are used by Lee et al.^6^ The use of the latter is because point‐level monitor data are not often dense at the small‐area scale of the disease data. This contrasts with studies quantifying the short‐term effects of pollution on health via a time‐series design, as these studies do not use small‐area data. The small‐area spatio‐temporal study design considered here poses a number of statistical modelling challenges that we now outline and subsequently address in this paper.

The first is how to construct a spatially representative pollution concentration for each areal unit, using both the point‐level monitoring and grid‐level modelled pollution concentrations. The point‐level measured data are typically spatially sparse (see, eg, Section 2), while the modelled concentrations, such as those produced by the atmospheric dispersion model developed by AEA Technology plc (AEA)^8^ used here, provide complete spatial coverage of the study region. However, the modelled concentrations are inherently less accurate than the monitoring data, as they are outputs from a model rather than real‐data measurements. As previously mentioned, existing health studies have used either data source in isolation to estimate areal‐level pollution summaries, but more recently fusion modelling (described in Berrocal et al^9^) has been proposed using both data sets for pollution prediction purposes (see, eg, Berrocal et al, Fuentes and Raftery, and McMillan et al(9, 10, 11)). Thus, in this paper, we propose a fusion‐based approach using both data sources to estimate areal unit–level pollution concentrations, which are then used in a health model.

The second modelling challenge is exposure uncertainty as argued by Blair et al,^12^ as the areal‐level pollution predictions produced from the pollution data are only estimates of the true spatially varying concentrations. A number of approaches have been proposed to incorporate pollution uncertainties and measurement errors into the health model, including Gryparis et al,^13^ Lee and Shaddick,^14^ Chang et al,^15^ Szpiro et al,^16^ and Szpiro and Paciorek.^17^ Most of these approaches have been set within a 2‐stage modelling paradigm. In this setting, the first stage comprises a spatio‐temporal pollution model for making predictions at unmeasured locations, while the second stage uses these predictions in a health analysis. Blangiardo et al^7^ make a number of pollution predictions for each areal unit and then fit the health model separately for each prediction set, before combining the estimated health effects. In contrast, Lee et al^14^ feed the entire variation in the predictive pollution distributions into the health model, while Chang et al^15^ consider the set of pollution predictions as a prior distribution in the disease model. Recently, Lee et al^18^ compare these approaches on data from England and find that the results depend on the amount of exposure uncertainty relative to the amount of spatial variation across the areal units.

The third modelling challenge we address is how to simultaneously estimate the joint health effects of multiple pollutants, as the air we breathe contains a complex mixture of correlated particle and gas phase pollutants (eg, nitrogen dioxide [NO_2_] and particulate matter less than 10 microns in size [PM_10_]), and these compounds depend on where we live. Existing approaches for addressing this issue include co‐pollutant models (eg, Yu et al^19^), constructing a composite air quality index (eg, Powell and Lee^20^), principal components decomposition (eg, Arif and Shah^21^), Bayesian kernel machine regression (eg, Bobb et al^22^), and Bayesian profile regression (eg, Coker et al^23^). However, each of these approaches has their limitations, including the impact of multicollinearity, the ad hoc attribution of weights to each pollutant, and the choice on the number of principal components to include in the health model. In this paper, we consider 2 pollutants, namely, NO_2_ and PM_10_, because they are the only ones measured at a sizeable number of locations to make a spatial modelling approach feasible.

In this article, we propose a methodological approach to address these 3 challenges simultaneously, in the form of a 2‐stage multivariate space‐time Bayesian hierarchical model with inference based on Markov chain Monte Carlo (MCMC) simulation. The first stage is a multiple‐pollutant space‐time fusion model, which uses the correlation between pollutants to improve pollutant prediction compared with more common single‐pollutant models. A few papers have modelled multiple pollutants concentrations using the output of an air quality model, such as Berrocal et al,^24^ Rundel et al,^25^ and Crooks and Isakov.^26^ The second stage is a space‐time disease model, which can estimate the joint health effects of 2 pollutants while accounting for their exposure uncertainty. This methodology is motivated by a new study of the effects of particulate matter and nitrogen dioxide pollution on respiratory hospital admissions in Scotland between 2007 and 2011. The remainder of this paper is organised as follows. Section 2 provides the background to the study and a summary of the data. Section 3 outlines our proposed modelling approach, while Section 4 validates the methodology against existing alternatives. Section 5 presents the results of our study while Section 6 contains a concluding discussion.

## 2 MOTIVATING STUDY

The methodological development is motivated by a new study based in mainland Scotland (displayed in Web Appendix A) which has a population of around 5.2 million people, between 2007 and 2011. Data are available at a yearly resolution for *T*=5 years for *n*=1207 areal units called intermediate geographies (IGs), which have an average population of around 4300 people.

### 2.1 Disease data

The disease data comprise of the yearly numbers of admissions to nonpsychiatric and nonobstetric hospitals in each IG between 2007 and 2011 with a primary diagnosis of respiratory disease (International Classification of Disease version 10 codes J00‐J99) and are freely available from http://statistics.gov.scot. We denote *Y*_*kt*_ as the observed number of respiratory hospital admissions for the *k*th IG and *t*th year. As the number of admissions in an IG depends on its population size and demographic structure, we use age and sex as external variables to calculate the expected number of admissions in each IG based on standard hospital admission rates stratified by age (0‐14, 15‐24, 25‐34, 35‐44, 45‐54, 55‐64, 65‐74, 75‐84, 85+ years) and sex for the whole of Scotland. These rates can be obtained from the Information Services Division, which is part of the National Health Service in Scotland. The expected count for area *k* and year *t* is denoted by *E*_*kt*_ and is given by $E_{kt}=\Sigma_{j=1}^{J}N_{jkt}r_{jt}$, where *N*_*jkt*_ is the population in area *k* in strata *j* in year *t*, and *r*_*jt*_ is the rate of disease for strata *j* in year *t* in Scotland.

**Figure 1:**
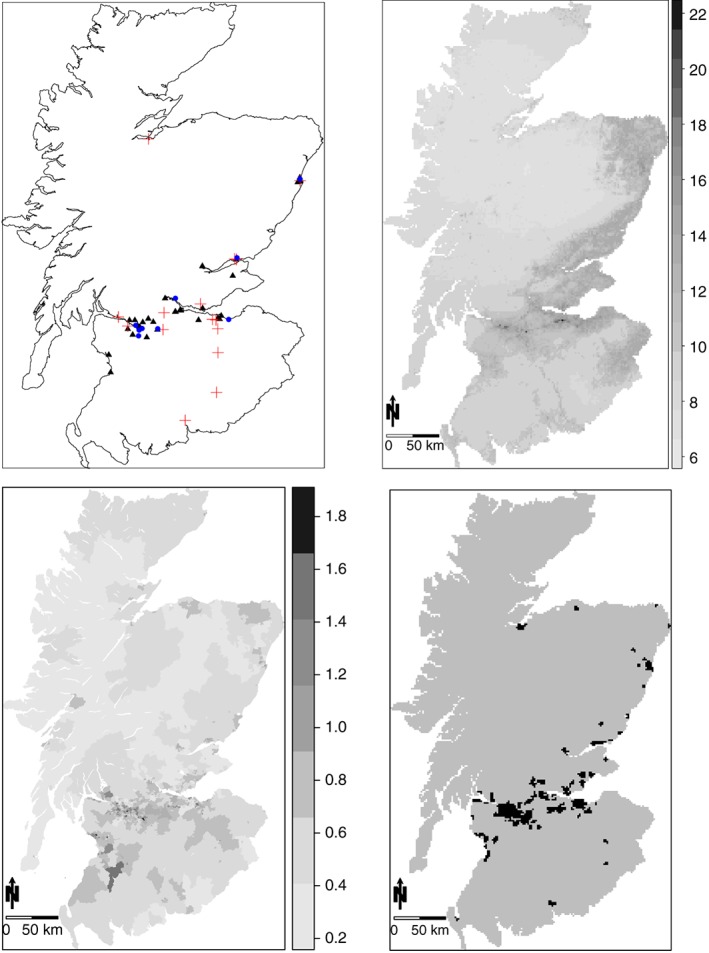
Summary of the data. Top left displays the monitoring sites for both NO_2_ and PM_10_ in 2010 ( ▴: common sites; +: sites with only NO_2_; •: sites with only PM_10_). Top right is a map of modelled annual average PM_10_ concentrations in 2010 (μ g/m ^3^). Bottom left is the standardised incidence ratio for respiratory disease in Scotland in 2011. Bottom right shows Scotland partitioned into urban (black) and rural areas (grey) [Colour figure can be viewed at http://wileyonlinelibrary.com]

An exploratory measure of disease risk is the standardised incidence ratio (SIR) given by *S* *I* *R*_*kt*_=*Y*_*kt*_/*E*_*kt*_, and an SIR of 1.1 indicates a 10*%* increased risk of disease compared to that expected. A spatial map of the SIR for 2011 is shown in the bottom left panel of Figure 1 and shows that the majority of the high‐risk IGs are in the major cities of Glasgow and Edinburgh, which are the set of small densely populated IGs in the lower middle part of the country (see Web Appendix A for a map of Scotland).

### 2.2 Pollution data

The pollutants considered in this study are NO_2_ and PM_10_, which are the only pollutants considered as the remainder were very sparsely monitored in Scotland during the study period precluding their inclusion in this study (see http://www.scottishairquality.co.uk). Data on annual mean concentrations between 2006 to 2010 are used rather than 2007 to 2011. This is so that the annual hospital admissions are related to pollution data from the preceding year, ensuring the pollution exposure occurred before the hospital admissions. We obtained 2 types of annual average pollution data, measured concentrations at a small number of locations and modelled concentrations at a 1‐km square resolution from the atmospheric dispersion model developed by AEA.^8^ The measured data are available at http://www.scottishairquality.co.uk/, while the modelled concentrations can be downloaded from https://uk‐air.defra.gov.uk/data/pcm‐data.

The top‐left panel of Figure 1 displays the locations of the monitoring sites for both NO_2_ and PM_10_ in 2010, from which it is evident that the monitors are mostly located in the cities, particularly in Glasgow and Edinburgh (see Web Appendix A for a map of Scotland). Figure 1 shows that not all sites measure both pollutants, and the 3‐dimensional rectangular array of observed concentrations over *m* sites for *q* pollutants for *T* time periods contains some missing values. We use the term “missing values” broadly, as there were many more pollution monitors operational in 2010 compared with 2006, resulting in a large amount of missing (not present) values in the earlier years. This situation is summarised in Table 1, which presents the numbers of monitors available in each year. The presence of these missing values causes problems with the model proposed in Section 3, and we treat each missing value as an unknown parameter in our Bayesian implementation of the model via a Markov chain Monte Carlo updating scheme. Details are given in Section 3.1.2. The monitor locations are clustered around the urban areas, with large rural areas having no pollution monitors (see Figure 1). This makes a geostatistical model for the monitored data inappropriate, as there are areas of the country whereby a potential prediction location would be a large distance from the nearest data point. This in turn will lead to large prediction errors and uncertainties, and a fuller description of this point can be found in Huang et al.^27^ Table 1 provides a summary of the monitoring data by year, pollutant, and site type, where the latter includes urban background, kerbside, roadside, and rural. Note that a kerbside station is within 1 m of the kerbside of a busy road, while a roadside station is located between 1 m of the kerbside of a busy road and the back of the pavement. Typically, this will be within 5 m of the road but could be up to 15 m. The table shows that concentrations recorded at urban locations are higher than those at rural locations as expected, and the number of rural monitors has remained almost unchanged while urban, kerbside, and roadside monitors have greatly increased over the 5 years duration of the study. Table 1 also shows the numbers of common sites for both PM_10_ and NO_2_. For example, in 2010 there are 48 monitoring sites measuring NO_2_ and 42 measuring PM_10_, among which 33 sites have measurements for both pollutants.

**Table 1:** Summary of the monitoring data by site type and year. The numbers within the round brackets represent the number of sites in the form (NO_2_and PM_10_), while those within square brackets indicate their corresponding mean concentrations ( μ g/m ^3^)

Site type	2006	2007	2008	2009	2010
Urban background	(3, 2)	(3, 3)	(6, 6)	(6, 6)	(6, 7)
	[27.3, 20.0]	[26.3, 17.0]	[27.0, 16.2]	[26.3, 14.1]	[26.0, 14.2]
Kerbside	(1, 1)	(4, 1)	(4, 1)	(3, 2)	(5, 2)
	[68.0, 38.0]	[64.0, 32.0]	[65.5, 27.0]	[67.3, 22.0]	[59.0, 24.0]
Roadside	(11, 8)	(15, 11)	(25, 20)	(30, 26)	(34, 32)
	[43.8, 24.1]	[42.4, 22.2]	[36.9, 20.8]	[36.2, 17.7]	[38.2, 19.2]
Rural	(3, 1)	(3, 2)	(3, 2)	(3, 1)	(3, 1)
	[8.0, 15.0]	[8.0, 10.5]	[8.3, 10.5]	[7.33, 11.0]	[9.33, 12.0]
Numbers of common sites	10	14	22	25	33

In addition to the monitoring data, we also have modelled concentrations at a 1‐km resolution, that were produced by AEA^8^ and available from the Department for Environment, Food and Rural Affairs (DEFRA). These modelled concentrations are outputs from the Pollution Climate Mapping model, which is a deterministic model that mathematically approximates the underlying physical and chemical processes via nonlinear partial differential equations, and the predictions are given in terms of averages over grid cells without any information about the inherent uncertainty. The combination of both data sets will allow us to better predict pollution than using the monitored data in isolation, as the modelled concentrations have complete spatial coverage of Scotland unlike the monitoring data. Modelled PM_10_ concentrations for 2010 are displayed in the top‐right panel of Figure 1, which shows again that the concentrations are much higher in the cities such as Glasgow and Edinburgh. The map of modelled NO_2_ concentrations has similar features and is not shown here (it can be seen in Huang et al^27^).

### 2.3 Covariate data

Covariate data are also available for both the pollution and disease models. For the pollution model, temperature is an important covariate, because it can affect air circulation and thus the spatial distribution of air pollution. Temperature data are available as annual averages across Scotland at a 5‐km resolution from the Met Office (http://www.metoffice.gov.uk/) and exhibit a general north‐south trend. For the disease model, socio‐economic deprivation is the major confounder, as populations that are more affluent exhibit better health on average due to factors such as lower smoking rates.^28^ However, socio‐economic deprivation is multidimensional and difficult to measure,^29^ as it is affected by a number of factors such as access to services, crime, education, skills and training, employment, and income. Therefore, here we have 2 proxy measures of socio‐economic deprivation. They are the percentage of people living in each IG who are in receipt of Jobseeker's Allowance (JSA), a benefit paid to working age people who are unemployed, and the median property price in an area (a natural log transformation is taken as it better fits the data and is denoted as log price). The percentage of people in receipt of JSA ranges between 0.05*%* and 15.3*%* with a median value of 2.7*%*, while the median property price in an IG ranges between £22,800 and £500000, with a median value of £125000.

Finally, for pollution prediction purposes, we use the Scottish Government urban and rural classification to split Scotland into urban and rural areas, which is shown in the bottom‐right panel of Figure 1. Note that the prediction locations will be the centres of the 68 448 one‐km grid squares on which the DEFRA concentrations are computed, and hence, they represent the average pollution concentrations in each 1‐km region. Therefore, we do not specify any of the locations as roadside or kerbside, as the majority of each grid square will not comprise just roads (there will of course be roads in a large number of grid squares). Therefore, we make a choice for each prediction location being urban background or rural.

## 3 METHODOLOGY

We propose a novel 2‐stage space‐time Bayesian hierarchical model for estimating the joint long‐term effects of multiple pollutants on health, while accounting for the uncertainty in the pollution concentrations when estimating their health effects. The first stage is a pollution fusion model, that models the pollution monitoring data in terms of the modelled concentrations and other covariates. This model is then used to predict pollution concentrations in each IG, so as to align with the disease data in stage 2. The pollution model extends the single pollutant model proposed by Huang et al^27^ to the multiple‐pollutant sphere. This multivariate approach uses the correlation between the 2 pollutants (NO_2_ and PM_10_ in our study) to improve the predictions of both, as both pollutants are not measured at the same set of locations. For example, consider predicting PM_10_ at a location that is close to a NO_2_ measurement but not close to a PM_10_ measurement. Then, using the correlation between (NO_2_ and PM_10_) should improve the PM_10_ prediction compared to a single‐pollutant model using PM_10_ data alone. The second stage is a disease model aiming to quantify the effects of the pollution concentrations estimated in stage 1 on the disease data. The disease model extends the model proposed by Rushworth et al^30^ in 2 ways, firstly by allowing for exposure uncertainty in the pollution concentrations when estimating their health impact and secondly by estimating the joint effects of multiple pollutants, simultaneously. Inference for this model is implemented within a Bayesian framework via MCMC simulation, and code to fit the model in the form of R functions is available from the first author on request.

### 3.1 Stage 1—pollution model

#### 3.1.1 Model specification

Observed pollution concentrations are available at *m* sites (the spatial locations of the sites are denoted by ***s***_*i*_, *i*=1,…,*m*) for *q* pollutants (here, *q*=2) for *T* consecutive years (here, *T*=5). Both the observed and modelled pollution data are modelled on the natural log scale because they are nonnegative and skewed to the right. Let $X_{j}^{\left(t\right)}={\left(X_{j}^{\left(t\right)}(s_{1}),\text{⋯},X_{j}^{\left(t\right)}(s_{m})\right)}^{\prime }$ denote the *m*×1 vector of monitoring observations (on the natural log scale) for the *j*th pollutant in year *t*, while ${\left(X_{1}^{\left(t\right)},\text{⋯},X_{q}^{\left(t\right)}\right)}^{\prime }$ denotes the extended vector for all *q* pollutants. The first level of our multivariate space‐time pollution model is given as

(1) $$\left[\begin{array}{c} X_{1}^{\left(t\right)}\\ X_{2}^{\left(t\right)}\\ ...\\ X_{q}^{\left(t\right)} \end{array}\right]∼N\left(\left[\begin{array}{ccc} Z_{1}^{\left(t\right)} & 0 & 0\\ 0 & ... & 0\\ 0 & 0 & Z_{q}^{\left(t\right)} \end{array}\right]\left[\begin{array}{c} \beta_{1}^{\left(t\right)}\\ \text{⋯}\\ \beta_{q}^{\left(t\right)} \end{array}\right],\sigma_{t}^{2}C_{q\times q}⊗I_{m}\right)\text{for}\;\;t=1,\text{⋯},T.$$

The mean of this regression model is given by $\left(Z_{1}^{\left(t\right)}\beta_{1}^{\left(t\right)},\text{⋯},Z_{q}^{\left(t\right)}\beta_{q}^{\left(t\right)}\right)$, comprising *m* × *p* design matrices $Z_{1}^{\left(t\right)},\text{⋯},Z_{q}^{\left(t\right)}$ and time‐varying coefficients $\left(\beta_{1}^{\left(t\right)},\text{⋯},\beta_{q}^{\left(t\right)}\right)$. The design matrices include a column of ones for the intercept term, the site type of monitoring station, the modelled concentrations (also on the natural log scale) and annual average temperature. Note that each monitoring site is assigned the closest gridded modelled concentration and temperature data. To account for the temporal autocorrelation in the data, the time‐varying regression coefficients (including the intercept term) are modelled by the following centred first‐order autoregressive process.

(2) $$\begin{array}{ccc} \beta_{i}^{\left(t\right)} & ∼N\left(\beta_{i}+\kappa (\beta_{i}^{\left(t-1\right)}-\beta_{i}),\tau^{2}\;I_{p\times p}\right)\;\;i=1,\text{⋯},q;t=2,\text{⋯},T, & \\ \beta_{i}^{\left(1\right)} & ∼N\left(\beta_{i},\tau^{2}I_{p\times p}\right),\\ \beta_{i} & ∼N\left(0,1000I_{p\times p}\right). \end{array}$$

The extent of the temporal dependence is captured by *κ*, which is assigned a uniform prior on the unit interval [0,1]. If *κ*=0, $\beta_{j}^{\left(t\right)}$ is smoothed towards a common parameter ***β***_*j*_ for all time periods, while if *κ*=1, $\beta_{j}^{\left(t\right)}$ is smoothed towards the parameters in adjacent years $\left(\beta_{j}^{\left(t-1\right)},\beta_{j}^{\left(t+1\right)}\right)$. The covariance matrix for the data in (1) is $\sigma_{t}^{2}C_{q\times q}⊗I_{m}$, where ***C***_*q*×*q*_ represents the between pollutant covariance at a common site, while the *m*×*m* identity matrix ***I***_*m*_ indicates that pollutants are assumed to be independent across space after covariate adjustment. The *i* *j*th element in ***C*** represents the covariance between pollutants *i* and *j* at each monitoring site for all time periods. The entire matrix ***C*** is assigned a weakly informative conjugate prior distribution, ***C***_*q*×*q*_∼,(*ν*=*q*,**Ψ**=100***I***_*q*×*q*_).

**Figure 2:**
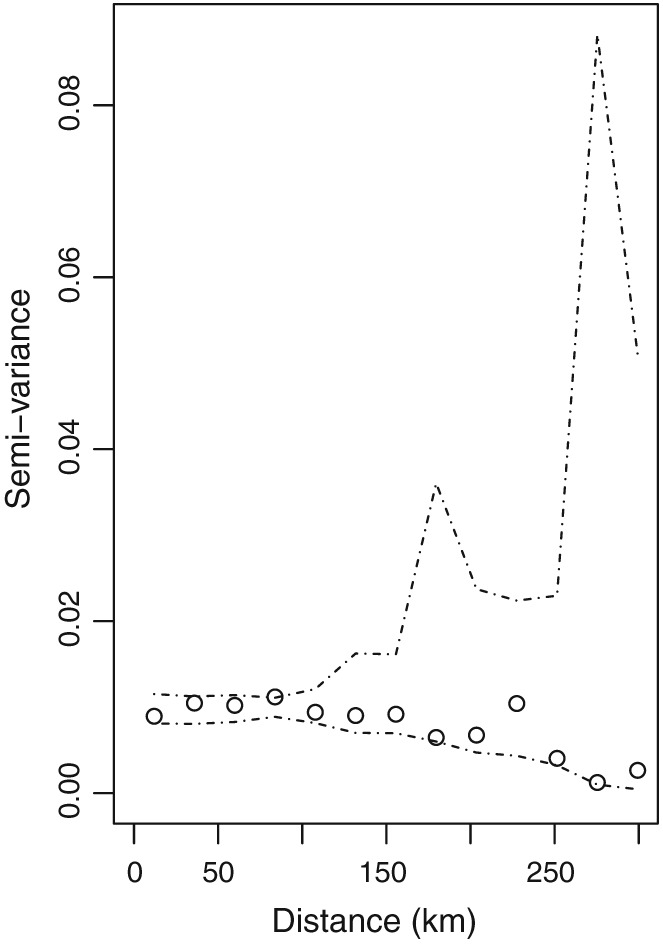
The empirical semivariogram of the residuals from a simple linear model for NO_2_ in 2010 (circles), with 95% Monte Carlo simulation envelopes (dashed lines) generated under the assumption of spatial independence

The assumption of spatial independence is appropriate because exploratory analysis suggests that after adjusting for the covariates in $Z_{j}^{\left(t\right)}$ no spatial autocorrelation remains. Specifically, we regressed the monitoring data against the modelled concentrations and the other covariates and examined the presence or absence of spatial autocorrelation in the residuals using semivariogram analysis. As an example, Figure 2 presents the empirical semivariogram (circles) for the residuals for NO_2_ in 2010, while 95% uncertainty intervals (dashed lines) based on the assumption of independence are constructed using Monte Carlo simulation. The figure shows that the empirical semivariogram lies within the 95% uncertainty intervals at all distances, suggesting that after accounting for the covariates, there is no remaining spatial autocorrelation that needs to be modelled. This is likely because the modelled concentrations (one of the covariates) are spatially smooth, which thus remove the spatial autocorrelation from the observed data. Semivariogram plots for the other years and for PM_10_ are similar and are not shown.

The parameter $\sigma_{t}^{2}$ is a scaling parameter to allow different levels of residual variation over time and is modelled as temporally autocorrelated via the following random walk prior on the log scale (as $\sigma_{t}^{2}$ must be nonnegative).

(3) $$\begin{array}{ccc} \ln(\sigma_{t}^{2}) & ∼N(\ln(\sigma_{t-1}^{2}),\delta^{2})\;t=2,\text{⋯},T, & \\ f(\ln(\sigma_{1}^{2})) & \propto 1. \end{array}$$

Finally, weakly informative conjugate inverse gamma (*a*=0.001,*b*=0.001) prior distributions are specified for the variance parameters (*δ* ^2^,*τ* ^2^).

#### 3.1.2 Missing values

As discussed in Section 2, not all sites measure both pollutants, and the number of sites with available data has increased over time. Therefore, the 3‐dimensional rectangular array of observed concentrations over *m* sites for *q* pollutants for *T* time periods contains some missing (not present) values. This missingness, broadly defined, is overcome by treating the missing data values as unknown parameters in the Bayesian hierarchical model and updating their values at each MCMC iteration based on the current values of the remaining parameters. Missing observations are updated separately for each site, and the full conditional distributions are obtained from standard conditional probability results for the multivariate Gaussian distribution.

#### 3.1.3 Pollution prediction and aggregation

The pollution model (1) is used to predict the concentrations of all *q* pollutants across mainland Scotland, where the prediction locations are the centres of the 1‐km grid squares at which the DEFRA concentrations are computed. This results in 68 448 prediction locations for each pollutant and year (*T*=5 years in total). After removing the burn‐in period of the MCMC run, we make *h* predictions at each prediction location for each pollutant and year combination, where here, *h*=100, which quantifies the posterior uncertainty in our predictions. Let $X_{tj}^{i}(s)$ denote the *i*th exponentiated prediction (as the measured data were modelled on the natural log scale) of the *j*th pollutant at location ***s*** and year *t*. Here, the disease data refer to irregularly shaped IGs, and we consider 2 different spatial aggregation metrics, mean, and maximum, for estimating IG‐level pollution concentrations. For the *i*th MCMC sample, these are computed as

(4) $$X_{ktj}^{i}=\frac{1}{N_{k}}\sum_{r\in A_{k}}X_{tj}^{i}(s_{r})\;\text{or}\;X_{ktj}^{i}=\max_{r\in A_{k}}\{X_{tj}^{i}(s_{r})\},\;i=1,\text{⋯},h,$$

where $A_{k}$ is the set of prediction locations that fall within the *k*th IG, while *N*_*k*_ is the cardinality of this set. If $A_{k}=\emptyset$, we use the closest prediction location for both metrics. We compare the mean and maximum metrics here, because the former is the commonly used metric in existing studies, while the latter represents peak concentrations within an IG. In IGs with mixed urban and rural components, the urban areas will be where the pollution is likely to be highest and where the majority of the people live, thus a spatial maximum may better capture average population exposure.

### 3.2 Stage 2—disease model

Recall that (*Y*_*kt*_,*E*_*kt*_) respectively denote the observed and expected numbers of disease cases in areal unit *k* during time period *t*. We denote ***b***_*kt*_ as the vector of associated covariates (JSA, log price) in areal unit *k* during time period *t*. Finally, $\left(X_{ktj}^{1},\text{⋯},X_{ktj}^{h}\right)$ denotes the sample of *h* predictions of the *j*th pollutant (using either the spatial mean or maximum, see Equation 4) for areal unit *k* and time period *t*. We first outline the baseline disease model that has previously been used to estimate the health effects of air pollution and then move on to describe our novel extension allowing for uncertainty in the pollution concentrations and the joint effects of multiple pollutants.

#### 3.2.1 Baseline disease model

The baseline model was proposed by Rushworth et al^30^ and assumes that the pollution concentration is not random and given by ${\bar{X}}_{ktj}=\frac{1}{h}\sum_{i=1}^{h}X_{ktj}^{i}$, which is the estimated aggregated pollution concentration obtained from the first‐stage model and estimated using the posterior mean. This model is given by

(5) $$\begin{array}{ccc} Y_{kt} & ∼\text{Poisson}(E_{kt}R_{kt}),\;k=1,\text{⋯},K,\;t=1,\text{⋯},T, & \\ \ln(R_{kt}) & =b_{kt}^{T}\alpha +{\bar{X}}_{ktj}\lambda +\phi_{kt},\\ \alpha & ∼N\left(0,1000I\right),\\ \phi_{t}|\phi_{t-1} & ∼N\left(\gamma \phi_{t-1},\nu^{2}Q{\left(\rho ,W\right)}^{-1}\right),t=2,\text{⋯},T,\\ \phi_{1} & ∼N\left(0,\nu^{2}Q{\left(\rho ,W\right)}^{-1}\right),\\ \lambda & ∼N(0,1000),\\ \nu^{2} & ∼\text{inverse gamma}\;(a=0.001,b=0.001),\\ \gamma ,\rho & ∼U[0,1]. \end{array}$$

The relative (to *E*_*kt*_) risk of disease in areal unit *k* and time period *t* is denoted by *R*_*kt*_ and is modelled on the log scale by covariates, ***b***_*kt*_, concentrations of a single‐pollutant ${\bar{X}}_{ktj}$, and a random effect *ϕ*_*kt*_. The regression parameters (***α***,*λ*) are assigned weakly informative zero‐mean Gaussian priors with a large diagonal variance matrix. The random effect *ϕ*_*kt*_ is included to allow for any residual spatio‐temporal autocorrelation remaining in the disease counts after the covariate effects have been accounted for. Here, ***ϕ***_*t*_=(*ϕ*_1*t*_,…,*ϕ*_*nt*_) denotes the vector of random effects for time period *t* and is modelled by a multivariate first‐order autoregressive process with temporal autocorrelation parameter *γ* and variance *ν* ^2^. Spatial autocorrelation is induced into the random effects by the precision matrix ***Q***(*ρ*,***W***)=*ρ*(diag(***W*** **1**)−***W***)+(1−*ρ*)***I***, which corresponds to the conditional autoregressive prior proposed by Leroux et al.^31^ The spatial dependence in the data is captured by a *n*×*n* neighbourhood matrix **W**, whose *i* *j*th element equals 1 if areas (*i*,*j*) share a common border and is 0 otherwise. The level of spatial autocorrelation in the random effects is controlled by *ρ*, and further details are given in Rushworth et al.^30^ Finally, weakly informative inverse gamma and uniform hyperpriors are specified for the parameters (*ν* ^2^,*ρ*,*γ*).

This baseline disease model (5) is deficient in 2 main ways. Firstly, it ignores the pollutant uncertainty by assuming the exposure ${\bar{X}}_{ktj}$ is not random and is instead known, and secondly, it only considers the effect of a single pollutant. Therefore, below, we extend this model to overcome both these issues.

#### 3.2.2 Allowing for pollutant uncertainty

To propagate the uncertainty in the pollution predictions into the health model, we propose a modified classical measurement error model for the predictions $\left(X_{ktj}^{1},\text{⋯},X_{ktj}^{h}\right)$. Letting *X*_*ktj*_ denote the true unknown exposure, then we extend the linear predictor in (5) to

(6) $$\begin{array}{ccc} \ln(R_{kt}) & =b_{kt}^{T}\alpha +X_{ktj}\lambda +\phi_{kt}, & \\ X_{ktj}^{i} & ∼N\left(X_{ktj},\sigma_{p}^{2}X_{ktj}^{2}\right),i=1,\text{⋯},h,\\ X_{ktj} & ∼N\left(\mu_{ktj},\sigma_{ktj}^{2}\right). \end{array}$$

A conjugate weakly informative inverse‐gamma prior is specified for $\sigma_{p}^{2}$ in common with the other variance parameters, while $\sigma_{ktj}^{2}$ is fixed to be large to also make this prior weakly informative. This model makes a number of assumptions about the predictions $\left(X_{ktj}^{1},\text{⋯},X_{ktj}^{h}\right)$, including unbiasedness, independence, and normality, as well as a quadratic relationship between the mean and variance of $\left(X_{ktj}^{1},\text{⋯},X_{ktj}^{h}\right)$ across IG and year combinations. Each of these assumptions is appropriate for the motivating Scotland study, and full details of the assumption checking are given in Web Appendix B.

#### 3.2.3 Estimating the joint effects of multiple pollutants

Model (5) allows one to estimate the effect of each pollutant separately, but it is desirable to estimate the joint effects of NO_2_ and PM_10_, simultaneously. The naive approach of putting both pollutants in the same model is inappropriate due to their high correlation (correlation of 0.74 in the measured point‐level data), which would lead to collinearity problems. Therefore, we propose including the first pollutant in the model, as well as the component of the variation in the second pollutant that is unrelated to the first pollutant. To compute the latter, we fit the following time‐varying linear regression model via least squares:

(7) $$X_{2}^{\left(t\right)}=\beta_{0}^{\left(t\right)}1+\beta_{1}^{\left(t\right)}X_{1}^{\left(t\right)}+\epsilon^{\left(t\right)}\;\epsilon^{\left(t\right)}∼N(0,\sigma^{2}I_{n\times n})\;t=1,\text{⋯},T,$$

where $X_{2}^{\left(t\right)}$ is a vector of the pollution data across all IGs for pollutant 2 at time *t*, while $X_{1}^{\left(t\right)}$ is a vector of the pollution data across all IGs for pollutant 1 at time *t*. Finally, ***ϵ*** ^(*t*)^ is a vector of errors across all IGs for time *t* while **1**=(1,…,1)_*n*×1_. The residuals from this model are denoted by ${\hat{\epsilon }}^{\left(t\right)}=X_{2}^{\left(t\right)}-{\hat{\beta }}_{0}^{\left(t\right)}1-{\hat{\beta }}_{1}^{\left(t\right)}X_{1}^{\left(t\right)}$, where $\left({\hat{\beta }}_{0}^{\left(t\right)},{\hat{\beta }}_{1}^{\left(t\right)}\right)$ are the least squares estimates of the regression parameters. These residuals ${\left({\hat{\epsilon }}^{\left(1\right)},{\hat{\epsilon }}^{\left(2\right)},\text{⋯},{\hat{\epsilon }}^{\left(T\right)}\right)}^{⊤}$ are uncorrelated with ${\left(X_{1}^{\left(1\right)},X_{1}^{\left(2\right)},\text{⋯},X_{1}^{\left(T\right)}\right)}^{⊤}$, and a proof of this is given in Web Appendix C. Thus, we extend the linear predictor in model (5) by

(8) $$\ln(R_{kt})=b_{kt}^{T}\alpha +X_{kt1}\lambda +{\hat{\epsilon }}_{kt}\lambda_{r}+\phi_{kt},$$

where, as before, a weakly informative Gaussian prior is specified for *λ*_*r*_. Here, ${\hat{\epsilon }}_{kt}$ is the residual variation in pollutant 2 not accounted for by pollutant 1 in area *k* and time *t*. However, in line with the previous section, we extend (8) to allow for uncertainty in ${\hat{\epsilon }}_{kt}$ using a similar measurement error approach. Specifically, given our *h* samples of each pollutant at each area and time period, we fit model (7) for each set of samples, yielding *h* sets of residuals $\left\{{\hat{\epsilon }}_{kt}^{i}\right\}$. These are then used in the following measurement error model:

(9) $$\begin{array}{ccc} {\hat{\epsilon }}_{kt}^{i} & ∼N\left({\hat{\epsilon }}_{kt},\sigma_{r}^{2}\right),i=1,\text{⋯},h, & \\ {\hat{\epsilon }}_{kt} & ∼N\left(\mu_{2kt},\sigma_{2kt}^{2}\right), \end{array}$$

where, again, weakly informative Gaussian and inverse gamma priors are specified for $\left({\hat{\epsilon }}_{kt},\sigma_{2kt}^{2}\right)$. We note that, here, we assume a constant measurement error variance unlike in (6), as this is suggested by the data (see Web Appendix B for full details).

## 4 MODEL VALIDATION

This section presents validation exercises for both the pollution model (Section 4.1) and the disease model (Section 4.2).

### 4.1 Pollution model validation

The pollution model proposed in Section 3.1 is compared to the single‐pollutant model proposed by Huang et al,^27^ which enables us to quantify the predictive advantages of a multiple‐pollutant modelling approach. We compare their performances via a leave‐one‐out cross validation exercise, which is applied to the subset of the sites that measure both pollutants in 2010. To perform the cross validation, we leave out a single‐pollution observation (either NO_2_ or PM_10_) at a single site in 2010 and use the remaining data to predict that value. This process is repeated for each pollutant and over all sites, and the bias, root mean square prediction error (RMSPE) and coverage probability of the 95% prediction interval are presented separately for each pollutant in Table 2. We only leave out sites in 2010 because it is the year with the largest data set to work with, as in earlier years, the pollutants were monitored at far fewer locations (see Table 1).

**Table 2:** Bias, root mean square prediction error (RMSPE) and 95% coverage probabilities for the 95% prediction intervals from a leave‐one‐out cross validation exercise for the single‐pollutant model (Huang et al^27^) and the multiple‐pollutant model (1)

Model	Bias	RMSPE	Coverage (%)
Single‐pollutant model for NO_2_	−0.008	0.248	96.6
Multiple‐pollutant model for NO_2_	−0.006	0.213	96.6
Single‐pollutant model for PM_10_	−0.015	0.160	90.0
Multiple‐pollutant model for PM_10_	−0.019	0.135	90.0

Note that Table 2 summarises the results from modelling the pollution data on the natural log scale. The table shows that both models produce largely unbiased results for both pollutants, with biases less than 0.019 in absolute value in all cases. This lack of substantial bias is likely to be because both pollution models calibrate the measured and modelled concentrations via intercept and slope parameters in Equation 1, thus having close to the correct pollution concentration on average. The key difference between the single‐ and multiple‐pollutant models is the RMSPE, with those from the multiple‐pollutant model being 14% and 16% lower for NO_2_ and PM_10_, respectively. This is mainly because the correlation between NO_2_ and PM_10_ is substantial and hence improves the prediction. Table 2 also shows that the RMSPE for NO_2_ is higher than for PM_10_, which is mainly because the variance/uncertainty in the NO_2_ observations is higher than that for PM_10_ (the estimated error variance for PM_10_ is 0.024 compared to 0.067 for NO_2_). Finally, Table 2 shows that both single‐ and multiple‐pollutant models have the same performance in terms of their coverages, which are close to the nominal 95% level for NO_2_. For PM_10_, the coverages are slightly lower at 90%, which is likely because the validation study is only based on 30 sites (two common kerbside sites and one common rural site are excluded in the validation study), and hence, coverage estimates will be unstable.

### 4.2 Disease model validation

The aim here is to quantify the impact on health effect estimation of ignoring or allowing for the measurement error in the pollution estimates when estimating their health effects. To this end, 2 variants of the disease model proposed in Section 3 are compared in this study. The first is the baseline model ignoring measurement error given by (5) and augmented by (7) and (8). Hereafter, this model is referred to as **BM**. The second model is the baseline model (5) augmented by (6), (7), (8), and (9), which allows for measurement error and is hereafter referred to as **UM**. We compare these 2 approaches by simulation, specifically focusing on the accuracy with which each approach can estimate the pollution‐health effect parameters *λ* (for NO_2_) and *λ*_*r*_ (for the residual variation in PM_10_ after being adjusted for NO_2_) on the relative risk scale. Four scenarios by specifying 4 differing levels of measurement error are considered in this simulation study. One hundred simulated data sets are generated under each scenario, then both models are applied to each simulated data set.

To generate each simulated data set initially, model **BM** is fitted to the real data with the spatial maximum aggregation metric. Here, (7) was used to estimate the residual variation in PM_10_ after NO_2_ adjustment. This yielded realistic values of the model parameters which are then fixed for all simulated data sets. Note that on the relative risk scales, this gave $\hat{\lambda }=1.030114$ and ${\hat{\lambda }}_{r}=1.005646$. The expected counts *E*_*kt*_ are also fixed at the values from the real data. Then, simulated disease count data are generated from the Poisson likelihood in model **BM** based on the above‐fixed quantities. However, the true pollution concentrations used to generate the simulated disease data are considered unknown, and instead, realisations are generated from (10), where the correlation between NO_2_ and PM_10_ is set to be 0.7 because it is similar to the real data (correlation between NO_2_ and PM_10_ in the measured point level data is 0.74).

(10) $$\left[\begin{array}{c} X_{kt1}^{i}\\ X_{kt2}^{i} \end{array}\right]∼N\left(\left[\begin{array}{c} X_{kt1}\\ X_{kt2} \end{array}\right],\sigma_{1}^{2}\left[\begin{array}{cc} X_{kt1}^{2} & 0.7X_{kt1}X_{kt2}\\ 0.7X_{kt1}X_{kt2} & X_{kt2}^{2} \end{array}\right]\right)\;k=1,\text{⋯},K;\;\;t=1,\text{⋯},T.$$

After each simulated data set (both pollution and disease) has been generated, models **BM** and **UM** are fitted separately, and the estimated relative risks are obtained. This process is repeated for each simulation data set in each scenario. When fitting model **BM**, a single realisation of $\left(X_{kt1}^{i},X_{kt2}^{i}\right)$ is generated and used, while when fitting model **UM** *h*=100 realisations are generated and used within the measurement error model. This simulation design thus mimics the situation when the true exposure is unknown, and either a single value or a set of values are used to represent it. We generate data under different levels of measurement error, with ${\hat{\sigma }}_{1}^{2}$ values of 0.001, 0.005, 0.01, and 0.05.

The results of the simulation are shown in Table 3, where the top panel (a) refers to the NO_2_ relative risk parameter denoted by *λ*, while the bottom panel (b) relates to the residual variation in PM_10_ after NO_2_ adjustment relative risk parameter denoted by *λ*_*r*_. In both cases, the table displays the bias, root mean square error (RMSE), width of the 95% credible interval (CI), and the coverage probabilities of these intervals. The results relate to the estimated relative risk (*λ*,*λ*_*r*_) of a 1 standard deviation increase in pollution, which is 6.84 *μ* *g*/*m* ^3^ for NO_2_ and 0.77 *μ* *g*/*m* ^3^ for residual PM_10_ after NO_2_ adjustment. Focusing on panel (a), the results show that the baseline model **BM** performs badly as the measurement error quantified by ${\hat{\sigma }}_{1}^{2}$ increases, showing increasing bias, RMSE, and much poorer coverage for its 95% CI. In contrast, model **UM** which accounts for pollution uncertainty shows much improved results, with the bias and RMSE being much smaller in absolute value than the corresponding results from model **BM**. For example, when ${\hat{\sigma }}_{1}^{2}=0.01$, the RMSE values are 0.0148 and 0.0035, respectively, showing around a 4‐fold increase for model **BM**. Additionally, the coverage probabilities are conservative throughout being above the nominal 95% level for model **UM**, which contrasts with model **BM** whose values tend to 0 as the amount of measurement error increases. Thus, the only downside of the measurement error model is that its uncertainty intervals are too conservative (wide) throughout.

**Table 3:** Simulation results for disease model validation in the form of bias, root mean square error (RMSE), and widths and coverages of the 95% credible intervals (CI). The results relate to top panel (a)—the relative risk (λ) of a 1 standard deviation increase in X_kt1_; bottom panel (b)—the relative risk (λ_r_) of a 1 standard deviation increase in ${\hat{\epsilon }}_{kt}$

Statistics	Model	${\hat{\sigma }}_{1}^{2}=0.001$	${\hat{\sigma }}_{1}^{2}=0.005$	${\hat{\sigma }}_{1}^{2}=0.01$	${\hat{\sigma }}_{1}^{2}=0.05$
(a)					
Bias	**BM**	−0.0013	−0.0089	−0.0143	−0.0251
	**UM**	0.0021	0.0025	0.0024	0.0012
RMSE	**BM**	0.0027	0.0096	0.0148	0.0251
	**UM**	0.0030	0.0035	0.0035	0.0028
CI width	**BM**	0.0267	0.0233	0.0198	0.0112
	**UM**	0.0290	0.0286	0.0287	0.0277
Coverage, %	**BM**	100	73	14	0
	**UM**	100	100	100	100
(b)					
Bias	**BM**	−0.0030	−0.0056	−0.0059	−0.0058
	**UM**	0.0008	0.0005	0.0002	−0.0015
RMSE	**BM**	0.0035	0.0058	0.0060	0.0059
	**UM**	0.0010	0.0010	0.0012	0.0025
CI width	**BM**	0.0114	0.0069	0.0052	0.0025
	**UM**	0.0152	0.0149	0.0147	0.0128
Coverage, %	**BM**	92	9	0	0
	**UM**	100	100	100	100

Table 3 also shows that the width of the 95% CI from model **BM** drops dramatically as ${\hat{\sigma }}_{1}^{2}$ increases, which corresponds to the increase in negative bias. This is because as the magnitude of the measurement error increases the single estimate of pollution used in model **BM** is less representative of the true pollution levels. As a result, no positive regression effect is estimated (ie, a regression coefficient close to 0), resulting in the negative bias seen in Table 3. The other consequence of this is that the model becomes more certain that there is no positive effect, resulting in a narrowing of the 95% CI with the increases of ${\hat{\sigma }}_{1}^{2}$. The bottom panel (b) in the table relates to the estimated relative risk (*λ*_*r*_) of a 1 standard deviation increase in residual PM_10_ effect after NO_2_ adjustment. Similar to panel (a), the table indicates that model **UM** outperforms model **BM** in terms of bias, RMSE, width of the 95% credible interval, and the coverage probabilities of these intervals.

## 5 RESULTS FROM THE SCOTLAND STUDY

Following the validation studies, we applied the full 2‐stage model proposed in Section 3 to data from the Scotland respiratory study, where exposure uncertainty was incorporated in (6) and (9), and both pollutants were included in the model following the approach in Section 3.2.3. The first‐stage pollution model was implemented once for predictive purposes, while the second‐stage disease model was implemented 4 times, 2 times for each spatial aggregation metric (spatial mean and spatial maximum). For each metric, the disease model was first run with NO_2_ and the residual variation in PM_10_ after adjusting for NO_2_(see Equation (7)), and then the roles of the 2 pollutants were reversed for the second run. In all cases, the model was run for 50 000 iterations, of which the first 20 000 were removed as the burn‐in period (after which convergence was assessed to have been reached). This resulted in inference being based on the remaining 30 000 posterior samples. From these samples, *h*=100 predictions were made of pollution at every 300th MCMC iteration, which, as shown in Web Appendix B, resulted in independent (over MCMC iterations) predictions.

The results of this study consist of 2 parts: arising from fitting the multivariate spatio‐temporal pollution model and then the disease model. The former is presented in Web Appendix D to save space, while the latter is presented here. The main results from fitting the disease models are shown in Table 4, where each column represents one of the model runs depending on the aggregation metric used and which pollutant was included first and which was included in its residual (after adjusting for the first pollutant) form. The first 3 rows present relative risks for each pollutant, where if NO_2_ was the pollutant included, then the residual pollutant was PM_10_. The remaining rows present the effects of the poverty‐related variables (log price and JSA) as well as the other variance and dependence model parameters. In all cases, the relative risks relate to a 1 standard deviation increase in each covariate, the values for which are presented at the bottom of the table.

**Table 4:** Posterior means and 95% credible intervals of the regression, autocorrelation and variance parameters from fitting the multiple‐pollutant disease model while allowing for exposure uncertainty. The regression parameters are presented as relative risks for a 1 standard deviation increase in each covariates value (see table note)

Parameter	Mean NO_2_	Max NO_2_	Mean PM_10_	Max PM_10_
Pollutant	0.992	1.034	1.014	1.033
	(0.979,1.002)	(1.021,1.046)	(1.003,1.024)	(1.024,1.043)
Residuals PM_10_	1.013	0.998	NA	NA
	(0.992,1.032)	(0.985,1.009)	NA	NA
Residuals NO_2_	NA	NA	0.978	1.012
	NA	NA	(0.954,1.004)	(1.004,1.024)
Log price	0.920	0.918	0.922	0.912
	(0.909,0.931)	(0.908,0.929)	(0.912,0.931)	(0.901,0.921)
JSA	1.202	1.193	1.194	1.186
	(1.183,1.217)	(1.175,1.208)	(1.179,1.209)	(1.171,1.203)
*ν* ^2^	0.061	0.060	0.061	0.059
	(0.056,0.065)	(0.056,0.065)	(0.056,0.065)	(0.055,0.063)
*ρ*	0.930	0.889	0.885	0.778
	(0.894,0.959)	(0.835,0.931)	(0.822,0.932)	(0.689,0.850)
*γ*	0.832	0.825	0.829	0.811
	(0.802,0.862)	(0.795,0.854)	(0.799,0.858)	(0.781,0.841)
$\sigma_{p}^{2}$	0.044	0.057	0.017	0.021
	(0.043, 0.044)	(0.057, 0.057)	(0.017, 0.017)	(0.021, 0.022)
$\sigma_{r}^{2}$	0.825	0.723	0.715	0.531
	(0.822, 0.828)	(0.721, 0.726)	(0.712, 0.717)	(0.529, 0.533)

Note: The standard deviation for NO_2_ is 6.84 *μ* *g*/*m* ^3^, PM_10_ 1.872 *μ* *g*/*m* ^3^, log price 0.38, JSA 2.35, residual mean PM_10_, max PM_10_, mean NO_2_, and max NO_2_ are 0.71, 0.77, 2.17, 2.61 *μ* *g*/*m* ^3^, respectively.

The main message from the table is that with the exception of the spatial mean NO_2_ metric, the other measures of pollution exhibit substantial long‐term effects on respiratory ill health, with relative risks ranging between 1.014 and 1.034. For both pollutants, the spatial maximum metrics exhibit larger health effects than the spatial mean metrics, with, for example, the relative risks for PM_10_ being 1.033 and 1.014, respectively, for the maximum and mean metrics. These increased effect sizes for the maximum metric may be because the spatial maximum better represents actual population exposure in each IG compared with the spatial mean. Consider an IG with both urban and rural components, then the spatial mean metric will average pollution concentrations over both components, whereas the spatial maximum metric will estimate exposure in just the urban component (which will likely have the highest concentrations). It is the urban component, where the majority of the population live, and hence, the spatial maximum is likely to be more representative of average exposure.

When considering the residual effects of the second pollutant after adjusting for the first pollutant, the epidemiological interpretation of the latter is not straightforward. As NO_2_ and PM_10_ are correlated, then they share some common spatio‐temporal variation. Thus, the variation in each pollutant can be partitioned into a pollutant‐specific component and a common component. Taking the spatial maximum as the aggregation metric, our results suggest that after accounting for NO_2_, the remaining component of PM_10_(ie, the PM_10_ specific variation) has no effect on respiratory hospitalisations. However, after adjusting for PM_10_, the remaining component of NO_2_ does exhibit a significant effect on respiratory hospitalisations. Collectively these results suggest that the component of variation in PM_10_ that is harmful to respiratory hospitalisations is also present in NO_2_, but that NO_2_ has an additional component harmful to respiratory health that is not present in PM_10_.

Both covariates measuring socio‐economic deprivation exhibit substantial effects on health that are largely independent of the pollution metric used, with increasing levels of poverty being associated with increased risks of hospital admissions. Additionally, the disease data show strong residual spatial and temporal autocorrelation after adjusting for the covariates, with the dependence parameters (*ρ*,*γ*) having posterior median values close to 1 in all cases. There is an existing literature on the impact of residual correlation on fixed effect estimates (see, for example, Hughes and Haran,^32^ and Lee and Sarran^33^) and the nature and extent of any biases depends on the spatio‐temporal similarity between the residual correlation and the fixed effects. The simulation study summarised in Table 3 suggests that in this study, there was not systematic bias, as all the biases are close to 0.

Finally, Table 4 shows that $\sigma_{r}^{2}$ is higher than $\sigma_{p}^{2}$ and the CI is wider, indicating that there is less certain information about $\hat{\epsilon }$ compared to ***X***_1_. The values for $\sigma_{p}^{2}$, which is the slope between var(*X*_1*kt*_) and $X_{1kt}^{2}$, indicate that the dependency between var(*X*_1*kt*_) and $X_{1kt}^{2}$ for NO_2_ is much stronger than PM_10_ as the slopes for NO_2_ are steeper than those for PM_10_.

## 6 DISCUSSION AND CONCLUSION

In this paper, we have proposed a novel 2‐stage Bayesian hierarchical space‐time model for estimating the long‐term health impact of air pollution. The model is novel in 3 main respects: (1) It has a multiple‐pollutant first‐stage fusion model that exhibits improved predictive performance compared with single‐pollutant models; (2) its second‐stage health model allows for the uncertainty in the estimated pollution concentrations when estimating their health effects; and (3) the second‐stage health model estimates the joint effects of 2 pollutants, simultaneously. The model is tested on real (first‐ stage model) and simulated (second‐stage model) data, before being applied to a new study of the relationship between respiratory‐related hospital admissions and NO_2_ and PM_10_ in mainland Scotland between 2007 and 2011.

From an epidemiological perspective, our key finding is that both NO_2_ and PM_10_ exhibit long‐term impacts on respiratory hospitalisation rates, although the former only exhibits an effect if the spatial maximum aggregation metric is used. These findings suggest that even though Scotland has relatively low levels of air pollution, the health effects persist. Our findings also suggest that the choice of spatial aggregation metric used to quantify areal‐level pollution concentrations has a major impact on the resulting health effect estimates, which naturally leads to the question of which metric should one use. The spatial mean is the commonly used metric in existing studies, but in IGs that contain both low (rural) and high population density (parts of cities or towns) areas, the spatial maximum concentration within an IG (likely the urban area) may be more representative of exposure as it represents where people actually live within that IG.

From a statistical perspective, we have shown the improvement in prediction that can be obtained from multiple‐ rather than single‐pollutant fusion modelling, and in the future, we will extend this work to consider study regions where a wider range of pollutants are measured at more than a handful of monitoring sites. We have also shown that allowing the exposure uncertainty to be propagated into the health model is important in epidemiological studies, because the predicted exposures are subject to errors and uncertainties. Given the existence of exposure variation, our simulation study shows that the estimated relative risks are attenuated if the exposures are assumed not to be random in the disease model. In contrast, the health model we propose that accounts for exposure uncertainty does not suffer from this problem, suggesting it is a valuable addition to the pollution‐health modelling toolkit.

Finally, we have shown how one can include 2 correlated pollutants in the same disease model while overcoming the issue of collinearity. The residuals from the temporally varying linear model are interpreted as the remaining signal from the second pollutant that is not explained by the first pollutant. Thus, the corresponding coefficient in the disease model represents the health effects resulting from this residual variation in the second pollutant that is not explained by the first pollutant. In this study, we fit the model twice with NO_2_ and then PM_10_ treated as the first pollutant, and the need to fit the model both ways around is a natural limitation of our approach. A further limitation is that extending the number of pollutants beyond 2 results in an even larger combination of different pollutant orderings, and in the future work, we will investigate this issue with the aim of creating a generalisation of the theory to *q*>2 pollutants that does not suffer from these problems. Another potential limitation of our work is the 2‐stage paradigm, which is standard practise in this setting. It is unclear what, if any, biases may arise from a 2‐stage approach, and in the future work, we will investigate comparing the approach presented here with a 1‐stage combined pollution and disease model, which will enable us to see the effect of cutting the feedback between the pollution and disease models.

## Supporting information

Supporting info item
