# Supplementary material for: Multivariate space‐time modelling of multiple air pollutants and their health effects accounting for exposure uncertainty
Source: Stat Med. 2017 Dec 4;37(7):1134–48. doi: 10.1002/sim.7570 (PMC5888175; doi:10.1002/sim.7570)
Supplement: Supplementary file 1 — Supporting info item [file SIM-37-1134-s001.zip › Supplementary.pdf]

Research Article

Statistics in Medicine

Received XXXX

(www.interscience.wiley.com) DOI: 10.1002/sim.0000

Supplementary material for "Multivariate space-time modelling of multiple air pollutants and their health effects accounting for exposure uncertainty"

Guowen Huang, *† **Duncan Lee and E. Marian Scott**

Introduction

This web based supplementary material has the following sections. Web Appendix A presents a map for the study region, while Web Appendix B provides evidence for the modelling assumptions made for the Scotland respiratory disease and air pollution study. Web Appendix C provides a proof of a result stated in the paper, and finally Web Appendix D provides results from the first-stage pollution modelling.

Web Appendix A: Exploratory analysis

Figure 1 displays a map of the mainland Scotland study region depicting the main cities and towns, which allows the locations of the data in the maps in this paper to be identified. Scotland has 4 main urban centres, Glasgow, Edinburgh, Aberdeen and Dundee, while the rest of the country is mostly rural.

Web Appendix B: Assumption validation

Recall that the classical measurement error model given by (6) in the main paper made a number of assumptions about the posterior predictive samples of the areal unit level concentrations $(X_{ktj}^1, ..., X_{ktj}^h)$ from the pollution model in stage 1. The first is that they are unbiased, which appears to be true from the negligible bias observed in the leave-one-out cross validation exercise conducted in Section 4 of the main paper (see Table 2 in the main paper). The second assumption is

School of Mathematics and Statistics, University of Glasgow, Glasgow G12 8SQ, UK
* *Correspondence to: School of Mathematics and Statistics, University of Glasgow, Glasgow G12 8SQ, UK*
† E-mail: hgw0610209@gmail.com

Copyright © 2017 John Wiley & Sons, Ltd.

Prepared using simauth.cls *[Version: 2010/03/10 v3.00]*

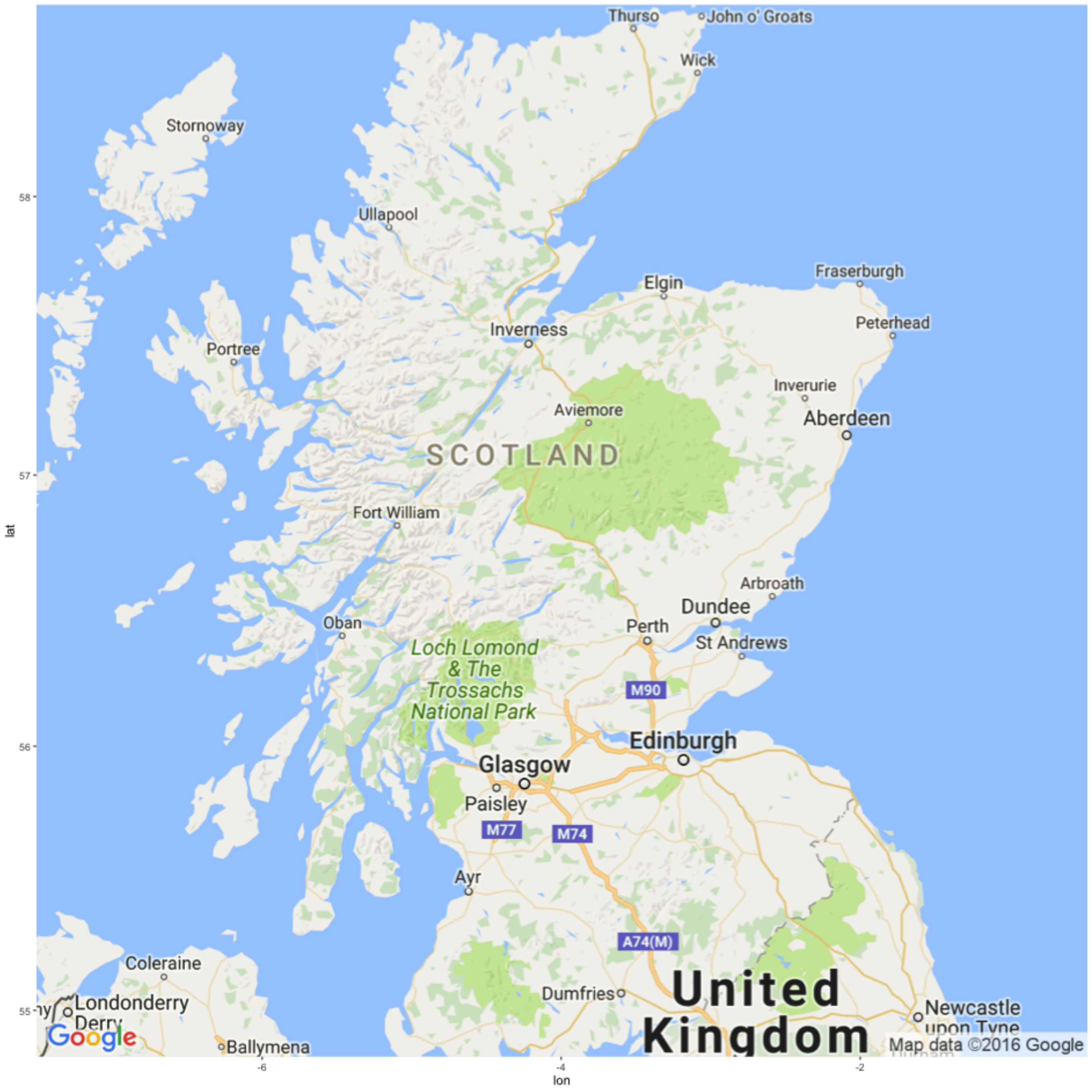

Figure 1. Map of Scotland showing a selection of the main cities and towns.

independence of the samples within a single Markov chain, which was ensured by only making a prediction at every 300th MCMC sample, yielding 100 predictions ($h = 100$) in total. This is illustrated in Figure 2, which shows the autocorrelation function of $(X^1_{ktj}, ..., X^h_{ktj})$ for a randomly selected (k, t) for both pollutants and both spatial mean and spatial maximum aggregation functions. In all cases the samples show no autocorrelation. The next assumption is normality, which is evidenced by the normal QQ-plots presented in Figure 3 for the same posterior predictive samples as the previous figure.

The last assumption of model (6) is the quadratic mean-variance relationship between the samples $(X^1_{ktj}, ..., X^h_{ktj})$ at different IG and time period combinations. This is evidenced by Figure 4, which plots the sample variance against the sample mean (left) and sample mean squared (right). Whilst a clear quadratic relationship appears in the left plot, a linear relationship is evident from the right plot based on the sample mean squared. The figure relates to NO_2 and the spatial maximum aggregation function, but similar results are found in the other cases.

Similarly, the constant variation assumption of $\{\hat{\epsilon}^i_{kt}\}$ in model (9) in Section 3.2.3 in the main paper is validated by

 Copyright © 2017 John Wiley & Sons, Ltd.

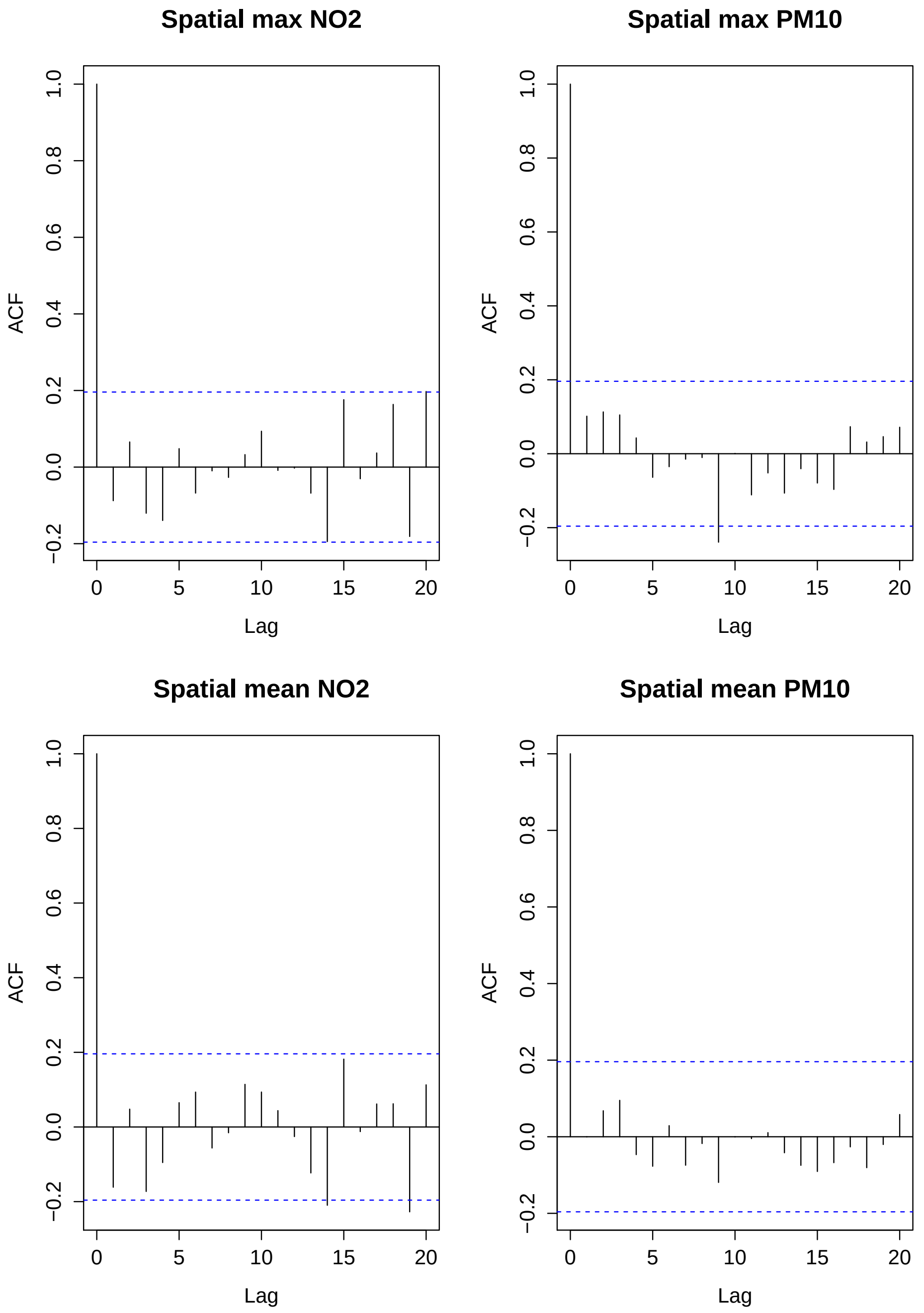

Figure 2. ACF plots for the sample $(X^1_{ktj}, ..., X^h_{ktj})$ for a randomly selected (k, t) for both pollutants and aggregation metrics.

Figure 5, which does not show the same mean variance relationship as the previous figure. The figure relates to the spatial maximum NO_2, but the plots for the other pollutants are similar and are not shown.

Web Appendix C: Proof of independence result

The following provides a proof of the independence between $\left(\hat{\boldsymbol{\epsilon}}^{(1)}, \hat{\boldsymbol{\epsilon}}^{(2)}, ..., \hat{\boldsymbol{\epsilon}}^{(T)}\right)^\top$ and $\left(\boldsymbol{X}_1^{(1)}, \boldsymbol{X}_1^{(2)}, ..., \boldsymbol{X}_1^{(T)}\right)^\top$ in Section 3.2.3 of the main paper.

Copyright © 2017 John Wiley & Sons, Ltd.

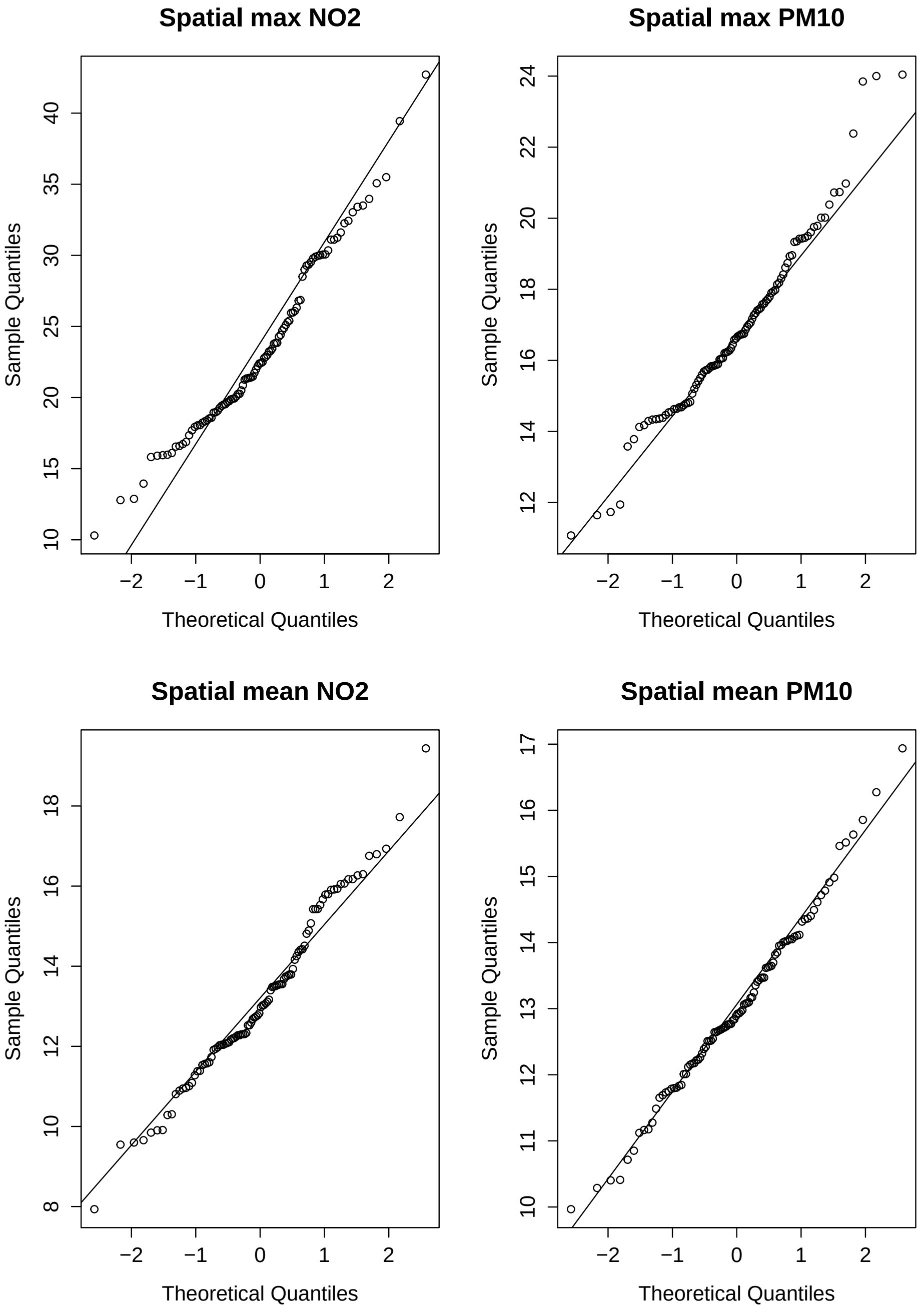

Figure 3. Normal QQ plots for the sample $(X^1_{ktj}, \ldots, X^h_{ktj})$ for a randomly selected (k, t) for both pollutants and aggregation metrics.

Proof

The estimated regression parameters $(\hat{\beta}_0^{(t)}, \hat{\beta}_1^{(t)})$ are obtained by least squares, by minimizing the sum of squared errors (SSE):

$$SSE(\hat{\beta}_0^{(t)}, \hat{\beta}_1^{(t)}) = \Sigma_{t=1}^{T}(\boldsymbol{X}_2^{(t)} - \hat{\beta}_0^{(t)}\mathbf{1} - \hat{\beta}_1^{(t)}\boldsymbol{X}_1^{(t)})^\top(\boldsymbol{X}_2^{(t)} - \hat{\beta}_0^{(t)}\mathbf{1} - \hat{\beta}_1^{(t)}\boldsymbol{X}_1^{(t)}).$$

Thus the estimates $(\hat{\beta}_0^{(t)}, \hat{\beta}_1^{(t)})$ were obtained by setting the partial derivatives of $SSE(\hat{\beta}_0^{(t)}, \hat{\beta}_1^{(t)})$ with respect to $\hat{\beta}_0^{(t)}$ and $\hat{\beta}_1^{(t)}$ equal to zero. Thus we have that

 Copyright © 2017 John Wiley & Sons, Ltd.

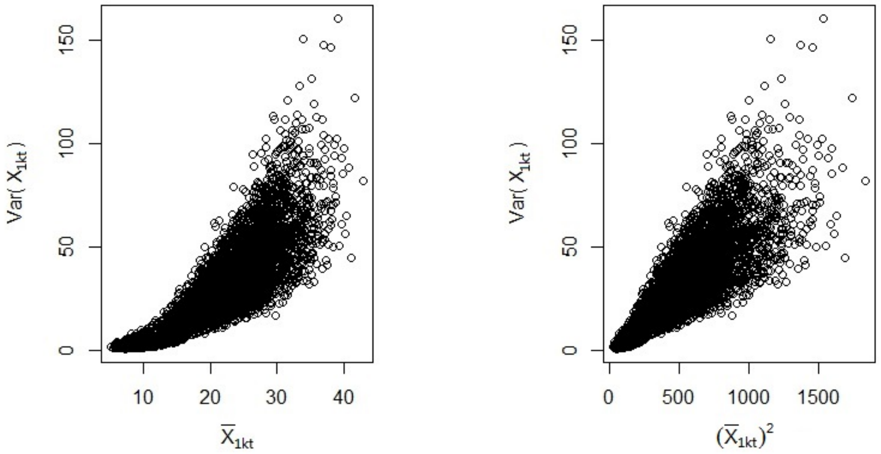

Figure 4. Scatter plots of the sample variance of $(X^1_{ktj}, \ldots, X^h_{ktj})$ against the sample mean (left plot) and the sample mean squared (right plot) for the maximum NO_2 aggregation metric.

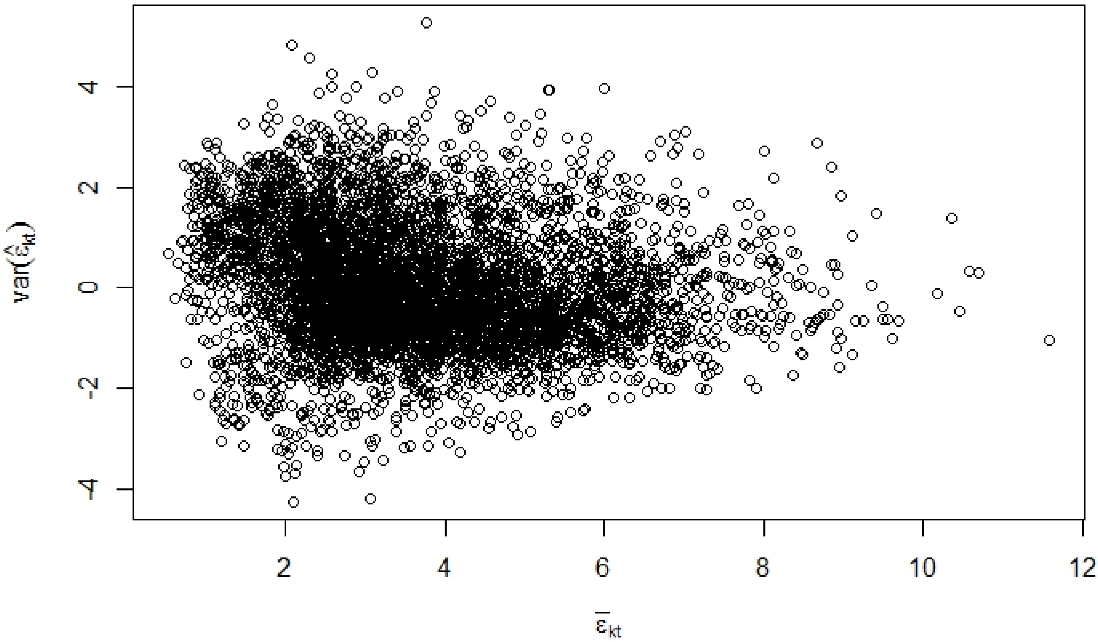

Figure 5. Scatter plot of the sample variance of $(\hat{\epsilon}^1_{kt}, \ldots, \hat{\epsilon}^h_{kt})$ against the sample mean for the maximum NO_2 aggregation metric.

$$\frac{\partial SSE(\hat{\beta}_0^{(t)}, \hat{\beta}_1^{(t)})}{\partial \hat{\beta}_0^{(t)}} = 0, \tag{1}$$

$$\Rightarrow -2 \times \mathbf{1} \cdot (\boldsymbol{X}_2^{(t)} - \hat{\beta}_0^{(t)} \mathbf{1} - \hat{\beta}_1^{(t)} \boldsymbol{X}_1^{(t)}) = 0,$$

$$\Rightarrow \mathbf{1} \cdot \hat{\boldsymbol{\epsilon}}^{(t)} = 0,$$

Copyright © 2017 John Wiley & Sons, Ltd.

$$\frac{\partial SSE(\hat{\beta}_0^{(t)}, \hat{\beta}_1^{(t)})}{\partial \hat{\beta}_1^{(t)}} = 0, \qquad (2)$$
$$\Rightarrow -2 \times \boldsymbol{X}_1^{(t)} \cdot (\boldsymbol{X}_2^{(t)} - \hat{\beta}_0^{(t)}\mathbf{1} - \hat{\beta}_1^{(t)}\boldsymbol{X}_1^{(t)}) = 0,$$
$$\Rightarrow \boldsymbol{X}_1^{(t)} \cdot \hat{\boldsymbol{\epsilon}}^{(t)} = 0.$$

Equation (1) and (2) can be combined to give $(\boldsymbol{X}_1^{(t)} - \bar{X}_1\mathbf{1}) \cdot \hat{\boldsymbol{\epsilon}}^{(t)} = 0$, where $\bar{X}_1 = \frac{\Sigma_{k=1}^{n}\Sigma_{t=1}^{T}X_{1kt}}{kt}$. Then as $\mathrm{E}(\hat{\boldsymbol{\epsilon}}^{(t)}) = \mathbf{0}$ we have

$$(\boldsymbol{X}_1^{(t)} - \bar{X}_1\mathbf{1}) \cdot (\hat{\boldsymbol{\epsilon}}^{(t)} - \mathrm{E}(\hat{\boldsymbol{\epsilon}}^{(t)})) = 0,$$

and therefore summing over t

$$\Sigma_{t=1}^{T}(\boldsymbol{X}_1^{(t)} - \bar{X}_1\mathbf{1}) \cdot (\hat{\boldsymbol{\epsilon}}^{(t)} - \mathrm{E}(\hat{\boldsymbol{\epsilon}}^{(t)})) = 0.$$

Thus dividing this by a constant yields the following correlation of zero result.

$$\begin{aligned} & \mathrm{cor}\left(\left(\hat{\boldsymbol{\epsilon}}^{(1)}, \hat{\boldsymbol{\epsilon}}^{(2)}, ..., \hat{\boldsymbol{\epsilon}}^{(T)}\right)^{\top}, \left(\boldsymbol{X}_1^{(1)}, \boldsymbol{X}_1^{(2)}, ..., \boldsymbol{X}_1^{(T)}\right)^{\top}\right) \\ = & \frac{\Sigma_{t=1}^{T}(\boldsymbol{X}_1^{(t)} - \bar{X}_1\mathbf{1}) \cdot (\hat{\boldsymbol{\epsilon}}^{(t)} - \mathrm{E}(\hat{\boldsymbol{\epsilon}}^{(t)}))}{\sqrt{\Sigma_{t=1}^{T}(\boldsymbol{X}_1^{(t)} - \bar{X}_1\mathbf{1}) \cdot (\boldsymbol{X}_1^{(t)} - \bar{X}_1\mathbf{1})\Sigma_{t=1}^{T}(\hat{\boldsymbol{\epsilon}}^{(t)} - \mathrm{E}(\hat{\boldsymbol{\epsilon}}^{(t)})) \cdot (\hat{\boldsymbol{\epsilon}}^{(t)} - \mathrm{E}(\hat{\boldsymbol{\epsilon}}^{(t)}))}} \\ = & 0. \end{aligned}$$

Web Appendix D: Results from fitting pollution model

The multi-pollutant spatio-temporal pollution model proposed in Section 3.1 was fitted to the yearly NO_2 and PM_{10} data between 2006 and 2010. Inference for the model was based on 50,000 MCMC iterations, of which the first 20,000 were removed as the burn-in period (after which convergence was assessed to have been reached). Table 1 displays the posterior means and 95% credible intervals of the time-varying regression parameters, where those with a 1 subscript relate to NO_2 and those with a 2 subscript relate to PM_{10}. The main result is that these estimates vary little over time, suggesting constant relationships between the covariates and measured pollution levels. The other main finding is that the size of the regression coefficient for the modelled data is higher for NO_2 than for PM_{10} (0.43 compared to 0.29), indicating a stronger relationship between modelled and observed concentrations for the former. Table 1 also validates the inclusion of temperature in the proposed pollution model as none of its 95% credible intervals contains 0.

The model was then used to predict the concentrations of both pollutants at a 1km resolution across mainland Scotland, which comprises 68,448 prediction locations for each year. From the 30,000 samples $h = 100$ predictions were made at every 300th MCMC iteration, which results in the predictions from the same Markov chain being independent. The predictions were random draws from the posterior predictive distribution of the concentrations at the prediction locations given the observed data, and were made separately at each site based on model (1), which in any event does not contain any spatial autocorrelation. Figure 6 displays these predictions aggregated to the IG level for 2010 by the spatial mean aggregation function, where the top row relates to NO_2 and the bottom row to PM_{10}. The left column is the posterior mean

Copyright © 2017 John Wiley & Sons, Ltd.

while the right panel is the posterior standard deviation. The maps show that unsurprisingly the concentrations for both pollutants are highest in the 4 largest cities (see Figure 1) and are relatively low in the rest of the country. The uncertainty is also largest in the cities, which is probably because in the rural countryside the concentrations are very low and therefore have little spatial variation and uncertainty.

Table 1. Posterior means and 95% credible intervals for the regression parameters from the multi-pollutant model. A 1 subscript relates to NO_2 while a 2 subscript relates to PM_{10}. The superscript denotes the year of the pollution data, with a 1 denoting 2006 and a 5 denoting 2010.

Parameter	$\boldsymbol{\beta}_1^{(1)}$	$\boldsymbol{\beta}_1^{(2)}$	$\boldsymbol{\beta}_1^{(3)}$	$\boldsymbol{\beta}_1^{(4)}$	$\boldsymbol{\beta}_1^{(5)}$
Intercept	1.14	1.14	1.14	1.14	1.14
	(0.83, 1.47)	(0.83, 1.47)	(0.83, 1.46)	(0.83, 1.46)	(0.83, 1.46)
Kerbside	0.88	0.88	0.88	0.89	0.88
	(0.74, 1.01)	(0.75, 1.01)	(0.75, 1.02)	(0.75, 1.02)	(0.75, 1.01)
Roadside	0.51	0.50	0.49	0.49	0.49
	(0.41, 0.61)	(0.41, 0.60)	(0.40, 0.59)	(0.40, 0.58)	(0.40, 0.59)
Rural	-0.73	-0.73	-0.72	-0.72	-0.72
	(-0.88, -0.57)	(-0.88, -0.57)	(-0.88, -0.56)	(-0.88, -0.56)	(-0.88, -0.56)
Modelled data	0.43	0.43	0.43	0.44	0.44
	(0.32, 0.53)	(0.32, 0.53)	(0.33, 0.53)	(0.34, 0.53)	(0.34, 0.54)
Temperature	0.09	0.09	0.08	0.08	0.09
	(0.05, 0.14)	(0.05, 0.13)	(0.04, 0.13)	(0.04, 0.12)	(0.04, 0.14)
Parameter	$\boldsymbol{\beta}_2^{(1)}$	$\boldsymbol{\beta}_2^{(2)}$	$\boldsymbol{\beta}_2^{(3)}$	$\boldsymbol{\beta}_2^{(4)}$	$\boldsymbol{\beta}_2^{(5)}$
Intercept	1.40	1.40	1.40	1.40	1.40
	(1.01, 1.93)	(1.01, 1.92)	(1.01, 1.93)	(1.01, 1.93)	(1.02, 1.93)
Kerbside	0.53	0.53	0.53	0.53	0.53
	(0.43, 0.64)	(0.43, 0.63)	(0.42, 0.63)	(0.42, 0.63)	(0.43, 0.63)
Roadside	0.29	0.30	0.30	0.30	0.31
	(0.22, 0.36)	(0.23, 0.36)	(0.24, 0.36)	(0.24, 0.36)	(0.25, 0.37)
Rural	-0.31	-0.31	-0.31	-0.31	-0.31
	(-0.43, -0.19)	(-0.43, -0.20)	(-0.43, -0.20)	(-0.43, -0.20)	(-0.43, -0.19)
Modelled data	0.29	0.28	0.29	0.29	0.30
	(0.13, 0.44)	(0.13, 0.43)	(0.13, 0.43)	(0.13, 0.43)	(0.14, 0.44)
Temperature	0.08	0.07	0.07	0.05	0.06
	(0.04, 0.12)	(0.04, 0.11)	(0.03, 0.11)	(0.01, 0.09)	(0.02, 0.10)

Copyright © 2017 John Wiley & Sons, Ltd.

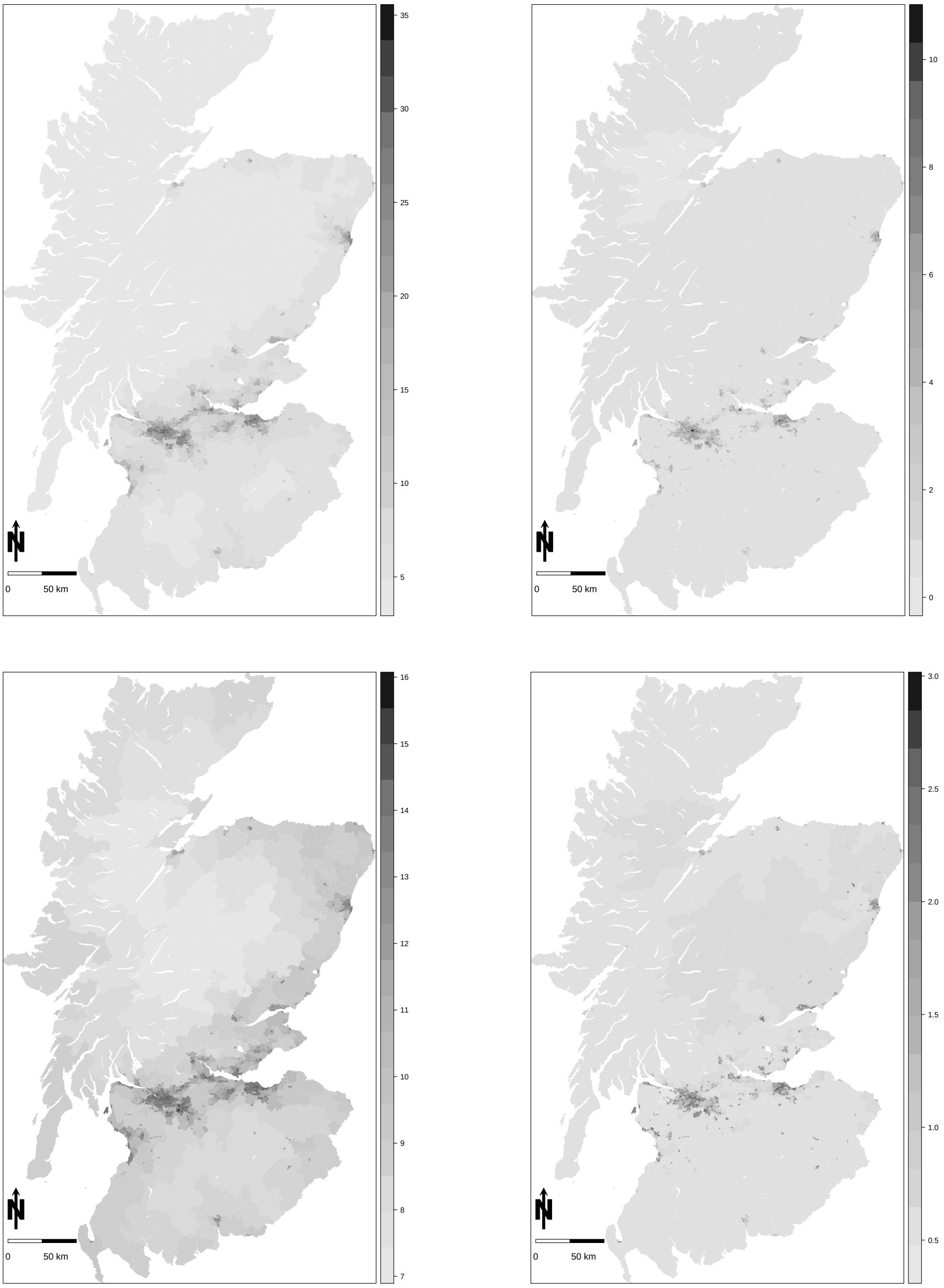

Figure 6. The predicted NO_2 and PM_{10} concentrations in $\mu g m^{-3}$ for 2010 at the IG scale. Top left panel is the spatial mean NO_2 concentration, while the top right panel is its posterior standard deviation. The bottom row relates to the spatial mean PM_{10}.

Copyright © 2017 John Wiley & Sons, Ltd.
